# Blending oxytocin and dopamine with everyday creativity

**DOI:** 10.1038/s41598-021-95724-x

**Published:** 2021-08-10

**Authors:** Anne Chong, Serenella Tolomeo, Yue Xiong, Dario Angeles, Mike Cheung, Benjamin Becker, Poh San Lai, Zhen Lei, Fabio Malavasi, Qianzi Tang, Soo Hong Chew, Richard P. Ebstein

**Affiliations:** 1 Department of Psychology, National University of Singapore, Singapore, Singapore; 2 Laboratory of Human Genetics, Department of Paediatrics, National University of Singapore, Singapore, Singapore; 3 MOE Key Laboratory for Neuroinformation, The Clinical Hospital of the Chengdu Brain Science Institute, University of Electronic Science and Technology of China (UESTC), Chengdu, China; 4 CCBEF (China Center for Behavior Economics and Finance), Southwestern University of Finance and Economics (SWUFE), Chengdu, China; 5 Department of Medical Science, University of Torino, Turin, Italy; 6 Fondazione Ricerca Molinette, Turin, Italy; 7 Farm Animal Genetic Resource Exploration and Innovation Key Laboratory of Sichuan Province, Sichuan Agricultural University, Ya’an, China; 8 College of Economics and Management, Zhejiang University of Technology, Hangzhou, China; 9 Department of Economics, National University of Singapore, Singapore, Singapore

**Keywords:** Transcriptomics, Behavioural genetics, Reverse transcription polymerase chain reaction

## Abstract

Converging evidence suggests that oxytocin (OT) is associated with creative thinking (CT) and that release of OT depends on ADP ribosyl-cyclases (*CD38* and *CD157*). Neural mechanisms of CT and OT show a strong association with dopaminergic (DA) pathways, yet the link between CT and *CD38*, *CD157*, dopamine receptor D2 (*DRD2*) and catechol-O-methyltransferase (*COMT*) peripheral gene expression remain inconclusive, thus limiting our understanding of the neurobiology of CT. To address this issue, two principal domains of CT, divergent thinking (AUT), were assessed. In men, both AUT is associated with gene expression of *CD38*, *CD157*, and their interaction *CD38* × *CD157*. There were no significant associations for DA expression (*DRD2*, *COMT*, *DRD2* × *COMT*) on both CT measures. However, analysis of the interactions of OT and DA systems reveal significant interactions for AUT in men. The full model explained a sizable 39% of the variance in females for the total CT score. The current findings suggest that OT and DA gene expression contributed significantly to cognition and CT phenotype. This provides the first empirical foundation of a more refined understanding of the molecular landscape of CT.

## Introduction

Creativity underpins the advancement of civilization and has driven the progress of *H. sapiens* from the middle Pleistocene to the current post-industrial Information Technology age. Among the most widely-applied definitions of creativity, the requirement for ideas that are both original and useful or appropriate stands out^1^. Creativity is a complex behaviour that is characterized by marked individual differences in cognitive flexibility and ability (exemplified by divergent thinking and problem solving)^2^ and open-mindedness, assessed in the Big Five personality trait of openness^3^.

With recent advances in molecular genetics, accumulated evidence suggests that creativity is highly heritable^4^. A more recent analysis shows that the genetic variance in creativity is explained partially by the genetic variance in intelligence and the personality trait of openness Like other complex traits, additive genetic effects and unique environmental factors play the major roles^1^. A recent study examined predictors for everyday creative activities^5^ and found openness to experiences and creative potential assessed by divergent thinking (DT) using the alternative uses test (AUT).

Several studies have tested specific polymorphic genes for a role in creativity^6,7^. These candidate gene studies, albeit small, especially highlight two neurotransmitter systems in contributing to creativity: oxytocinergic,dopaminergic and noradrenergic pathways. In an interesting review, Beversdorf reported that the noradrenergic system has an effect on performance on tasks associated with creativity^8^. Notably, the dopaminergic effects on creativity-task performance appear distinct from noradrenergic effects. In addition, De Dreu and colleagues^7,9^ found a role of OT in contributing to creativity by testing SNP associations within the OT receptor gene (*OXTR*) region using intranasal OT administration. In a literature review, they found mixed evidence for a role of *OXTR* SNPs in creative thinking^9^. Additionally, they also examined *CD38*, and in some but not all investigations, allele carriers of *CD38* SNPs scored lower on neuroticism and higher on imagination, personality traits related to creativity^12–12^.

CD38 is a type II transmembrane glycoprotein with ADP-ribosyl cyclase activity located both peripherally and in the brain^13^ that governs the central release of OT^14^. CD38 has numerous purposes acting as a receptor, ectoenzyme^15^ and second messenger performing as a ubiquitous calcium-signalling molecule^16^. Towards examining the role of neural gene pathways in contributing to CT (AUT), we examine gene expression for oxytocinergic (*CD38*, *CD157*) and dopaminergic (*DRD2*, *COMT)* gene expression in saliva samples and including Openness (NEOO), fluid intelligence (RPM), sex, and age in our model. *CD38* and its homologue *CD157* (BST-1), contiguous gene duplicates on human chromosome 4 (4p15), represent a gene family that regulates cellular interactions^15,17^. Importantly, an oxytocin analogue has long-lasting effects on anxiety behavior in a *CD157* knockout mouse^17^ and communication impairment during the suckling period is restored by oxytocin in the *CD157* knockout^18^. Altogether, studies with knockout mice reveal that *CD157*, as well as CD38, has a role in oxytocin (OT) release and both pivotally regulates social behavior^21^. *CD157* and *CD38* knockouts show decreased plasma oxytocin levels^22^. Consequently, we chose to examine both homologues in the current study.

Dopamine (DA) has been long considered to contribute to CT and two widely-studied polymorphic DA genes have been specifically linked to creativity, *DRD2*^6^ and *COMT*^21^ and recently integrated into the dual DA pathway model^23,24^.

Mesocortical DA projections to the forebrain are known to be involved in cognition^25^ and hence are also likely important in CT^24^. Notably, accumulating evidence suggests that these pathways are modulated by DA and OT^26,27^. A recent study reported the mechanisms of OT modulation of DA neurons using a combination of anatomical, optogenetic and electrophysiological approaches revealing the evidence of how the two systems interact contributing to the multi-faceted behavioural roles of OT^28^.

Drawing on these observations, in the current investigation, we sought to examine the role of oxytocinergic and dopaminergic pathways in contributing to creativity by using laboratory-based assessments of the two principal domains of creativity and divergent thinking (DT) measured by the alternative uses test (AUT)^29^. AUT is one of the most widely used assessments of domain-general DT cognition^31^.

Among the four components of DT that are evaluated by the AUT (i.e. fluency, flexibility, originality and elaboration) defined by Guilford^32^ only originality was used for the scoring in this study.

Originality is defined as the novelty or infrequency of ideas. The use of originality as a measure of novelty in the AUT is well-supported^33^. Insight problems require solutions that are apparently spontaneous and often attributed to unconscious and associative processes^34^.

To provide an important benchmark and increase the discriminant validity^35^ of the laboratory measures of CT, as well as to avoid critically confounding effects of intelligence and openness (see also^36^), the personality trait of openness (NEOO) measured using the NEO-PI-3^3^ and fluid intelligence^37^ indexed by the short version of Raven’s Standard Progressive Matrices^38^ (RPM) were additionally assessed in the present study.

The underlying mechanisms by which neural gene pathways contribute to CT (AUT) are undoubtedly complex. Here, we examine gene expression for oxytocinergic (*CD38*, *CD157*) and dopaminergic (*DRD2*, *COMT)* gene expression in saliva samples. Additionally, we included NEOO, RPM, age and sex in our model as many effects of oxytocin are sex-dependent, e.g^44–47^. Crucially, gene expression that captures both genetic and environmental information appears to have greater predictive power than the current genome.

## Methods

### Participants

200 students (101 females, *M*_age_ = 22.33, *SD* = 1.18) of Han Chinese descent from the National University of Singapore were recruited via an online advertisement in campus-wide student forum and were reimbursed $20 for their participation. All experimental protocols were approved by the Institutional Review Board of the National University of Singapore and each participant gave informed consent. Participants were inventoried on the Creativity measures of AUT, NEOO, RPM, *CD38*, *CD157*, *DRD2* and *COMT* gene expressions were included in this study. Two participants had invalid responses for the CT measures and were excluded bringing the final number of participants included in this study to 198 (101 females). However, there were incomplete measures of all 4 gene expressions, NEOO and RPM for some participants in various combinations of missingness. Therefore, the subsequent analyses are conducted within subsets of the participant pool that consisted only of participants with complete measures of the relevant variables, thus maximizing the sample size for each analysis.

### Creativity assessment

Creativity was assessed using the AUT and IPS (IPS was measured but a restriction of range in the outcomes prevented meaningful analyses and conclusions.) administered in a randomized order. AUT^48^ measures divergent thinking and comprises four measures of divergent thinking specifically originality, fluency, flexibility and elaboration. Notably, we define divergent thinking as the cognitive process used to generate creative ideas by exploring different solutions.

For this study, we scored only for the originality component i.e. uses that are highly novel, that is an established indication of creativity^2,49^. Participants were instructed to give as many unconventional uses as possible for three objects randomly chosen for the list of objects used in AUT (button, nail and pencil) within 6 min for all three objects following the methology described in Guildford^48^. Norming of each object was done within the study sample. A use that had frequency less than 2% were considered highly novel and given 2 points (e.g. using pencil to play pick-up sticks). Uses with frequencies between 2 and 4% were given 1 point and uses that were given by more than 4% were given 0 point (e.g. using a nail to hang a picture on the wall). The AUT score (*M*_AUT_ = 8.85, *SD* = 7.03) was a summation of scores for all three objects. For statistical analysis, each item score was standardized separately and then summed for a standardized AUT score per participant. This is to ensure items with larger means do not dominate the final AUT score.

For personality, participants were inventoried on their degree of openness to experiences using the Openness subscale of NEO-PI-3. The subscale examines facets such as fantasy, feelings, ideas, actions, aesthetics and values and consist of 48 items on a 5-point Likert scale. The mean score is 162.71 and *SD* = 18.43. The internal consistency is 0.88.

Fluid intelligence was measured using the 9-item Abbreviated Form of the Raven’s Progressive Matrices^38^with an internal consistency of 0.8^38^ which participants completed within 5 min. The total score with mean 6.69 and *SD* 1.78, is the sum of all correct answers. All analyses were conducted with Stata 12 using standardized values of the AUT. P-values adjustment for multiple models was carried out using the p.adjust function in R based on the Benjamini–Hochberg (BH) method for controlling the False Discovery Rate^50^.

### Saliva collection and gene expressions

#### Collection and processing of saliva

Samples were collected according to the manufacturer’s protocol (DNA Genotek Inc., Ontario, Canada). Participants rinsed their mouth with clean water and abstained from food or drink for 1 h before collection. About 2 ml of the whole saliva was deposited into the Oragene RE-100 tubes with preservative and stabilizer solution was derived by simulated habitual chewing for 2 min. The sample was reacted with an in-cap stabilizer solution which was released upon cap replacement. The preserved saliva solution was neutralized in 1/25th volume of Oragene RE-L2N solution on ice for 10 min before clearance by centrifugation for 5 min at 13,000 RPM.

#### RNA extraction from saliva

The extraction of RNA was performed using the RNeasy Micro kit (Qiagen, Hilden, Germany). Briefly, 500 μl of sample solution was mixed with 2 volumes of 95% ethanol and precipitated on ice for 30 min. The pellet was collected by centrifugation, completely dissolved in the RLT buffer to lyse the cells, then mixed with 70% ethanol. Nucleic acid was captured by centrifugation in a spin column followed by in-column treatment with DNase I for 15 min at room temperature. The sample was subjected to series of RPE buffer washes before final resuspension in 25 μl of RNase-free water. The quality and quantity of sample yield were assessed spectrophotometrically in the NanoDrop 2000 (Thermo Fisher Scientific, Waltham, MA).

### Real-time RT-PCR analysis

Gene expression was determined by qPCR in a 2-gene custom RT2 Profiler PCR array system (Qiagen) according to the manufacturer’s protocol. In the thermocycler, residual genomic DNA in 150 ng of total RNA sample was first treated in the genomic DNA elimination mix at 42 °C for 5 min, followed by the addition of reverse transcription mix and further incubation at 42 °C for 15 min. The template was added to the reaction mixture containing SYBR Green 1 Master mix with HotStart DNA polymerase which was then distributed to the PCR array at 25 μl per well. PCR was performed in the CFX96 qPCR detection system (Bio-Rad Laboratories, Hercules, CA) at cycling conditions that include an initial activation at 95 °C for 10 min followed by 40 cycles of annealing and extension steps at 95 °C for 15 s and at 60 °C for 1 min respectively. A final default melting curve program was applied to generate a first derivative dissociation curve for each well. The expression levels were analysed using the ΔCT method from Cq values normalized to the expression of select reference from a series of candidate genes. All methods were carried out in accordance with relevant guideline and regulations.

## Results

### Descriptive statistics

Table 1 shows the descriptive statistics of the study participants (n = 198) and the various measures including oxytocinergic and dopaminergic gene expressions. Initial t-tests show that females are younger than males (*p* < 0.001), likely since males are required to serve in the military before entering university. There are significant gender differences for RPM, with males scoring higher (*t*(195) = 2.92, *p* = 0.004), and *CD38* expression (*t*(162) = 3.05, *p* = 0.003). A meta-analysis of RPM shows that among adults, the male advantage is 0.33 *SD* units^51^. This sex difference in RPM appear likely to be influenced by sex differences in spatial ability^52^. There are no significant differences between sex and measures of AUT, NEOO, or the gene expressions of *CD157*, *DRD2*, *COMT* (all *p* > 0.05).

**Table 1:** Descriptive statistics for participants in the current study.

Sex	AUT	NEOO	RPM	CD38	CD157	DRD2	COMT	Age
**Male**								
*M*	9.59	162.77	7.06	1.87	0.85	1.91	1.66	22.33
*SD*	7.46	19.89	1.67	0.38	0.80	0.86	0.49	1.18
n	97	96	96	80	79	80	92	97
**Female**								
*M*	8.14	162.65	6.34	1.60	0.82	2.08	1.74	21.35
*SD*	6.55	17.02	1.81	0.63	0.87	0.84	0.50	1.01
n	101	101	101	84	66	75	92	101
**All**								
*M*	8.85	162.71	6.69	1.73	0.84	1.99	1.70	21.83
*SD*	7.03	18.43	1.78	0.54	0.83	0.85	0.50	1.20
n	198	197	197	164	145	155	184	198

Mean (*M*) and standard deviation (*SD*) of creative thinking, NEO-Openness (NEOO), Raven’s Progressive Matrices (RPM), age, and logged normalized values of *CD38*, *CD157*, *DRD2* and *COMT* gene expressions.

Creativity is indexed by Alternate Use Task (AUT) and Insight Problem Solving (IPS) tasks.

Total participants = 198 with n participants with available data for each respective measurement.

### Association between creativity score, RPM and openness

**Figure 1:**
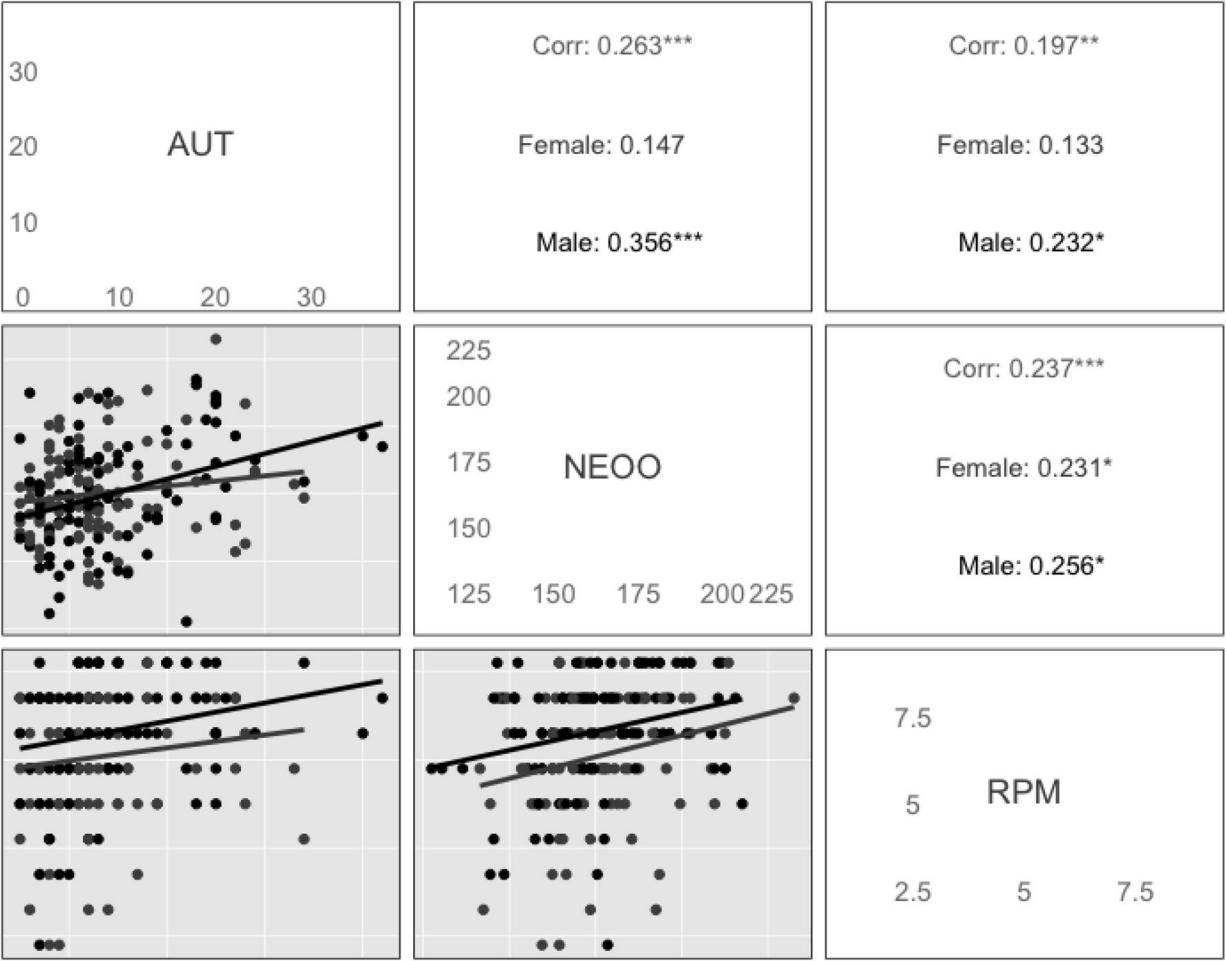
Matrix for pairwise correlations of AUT, NEO and RPM with coefficients for males and female were pooled (Corr) together and males and females separately. ***p < 0.01, **p < 0.05, *p < 0.1.

We first examine the correlations between creativity, fluid intelligence and personality. Figure 1 shows the pairwise correlation plots and the correlation coefficients. As expected, there are significant correlations at *p* < 0.05 between AUT and NEOO (r = 0.27), as well as with the RPM (r = 0.20), in the expected direction. Additionally, the NEOO was significantly correlated to RPM (r = 0.24).

### Regression analyses

Due to varying success in gene expression measurements which led to participants with different combinations of available data, three separate analyses were carried out to maximize sample sizes. Relationships between the AUT and gene expressions were first investigated within the oxytocinergic (Analysis 1, n = 133) and dopaminergic (Analysis 2, n = 147) systems separately and in the final analysis investigated interactions between the 2 systems (Analysis 3, n = 108). We investigated interactions of *CD38* and *CD157* within the oxytocinergic system (Analysis 1) because these two neurotransmitters regulate the release of central oxytocin and their interaction has been found to contribute to social behaviors^53^.

Gene–gene interaction analysis of *DRD2* and *COMT* single nucleotide polymorphisms (SNP) with significant 4-way and 3-way interactions were found to contribute to creativity^54^. Analysis 2 could validate and extend these SNP interactions findings in the dopaminergic system by directly analysing the expression products of the dopaminergic genes.

Finally, in Analysis 3, we investigated the interactions of the oxytocinergic and dopaminergic systems which have been known to play a role in social behaviors, personalities as well as disorders such as autism, addiction and depression.

### Analysis 1: relationship between CT assessments and ADP-ribosyl cyclase gene expression

To test the association between *CD38*, *CD157* gene expressions and their interactions with sex (focal independent variables) and creativity (the dependent variable), we carried out a linear regression analysis (OLS) with robust standard error controlling for age, NEOO and RPM in the model (Table 2). Model 1, pooling males and females together, is significant for AUT (F (10,122) = 2.52, R^2^ = 0.19, BH adjusted *p*_*adj*_ = 0.011). There is a significant effect of CD157 expression (*coef*. = − 4.70, *t*(122) = − 2.16, *p* = 0.03). However, there is a significant interaction effect of *CD157* and sex (*coef*. = 7.11, *t*(122) = 2.17, *p* = 0.03). In addition, the control variable NEOO significantly correlates with AUT (*coef*. = 0.01, *t*(122) = 3.12, *p* = 0.005). There is a marginally significant interaction between CD38 and CD157 (*p* < 0.08).

**Table 2:** The Relationship between oxytocinergic gene expression, openness and fluid intelligence on creative thinking.

Variables	Model 1	Model 2	Model 3	Model 4
	All	Male	Female	Male
CD38	0.84	1.02	1.73	
	(1.82)	(1.85)	(2.02)	
CD157	− 4.70*	− 4.68*	1.74	
	(2.17)	(2.12)	(2.42)	
CD38 × CD157	2.43^a^	2.38^a^	− 0.93	
	(1.35)	(1.32)	(1.28)	
Sex	− 2.16			
	(4.79)			
Sex × CD38	1.35			
	(2.71)			
Sex × CD157	7.11*			
	(3.28)			
Sex × CD38 × CD157	− 3.69^a^			
	(1.89)			
Age	0.32^a^	0.60**	− 0.11	0.58*
	(0.18)	(0.21)	(0.29)	(0.23)
NEOO	0.03**	0.03*	0.02	0.04**
	(0.01)	(0.01)	(0.02)	(0.01)
RPM	0.17	0.26	0.11	0.29*
	(0.12)	(0.14)	(0.20)	(0.13)
Observations	133	74	59	74
R-squared	0.19	0.31	0.05	0.19

Control variables used are age, openness and RPM.

**p < 0.01, *p < 0.05, ^a^*p* < 0.1.

Many effects of oxytocin are sex-dependent^39,40^ and oxytocin’s evolutionary function in mammals is especially crucial to female reproductive behavior, viz. helping regulate parturition and lactation. Moreover, in our first analysis we observe that the strength of the correlations between AUT, RPM and NEOO are moderated by sex. Hence, we subsequently conduct separate regression analyses for males and females.

When the participants are stratified by sex: males (Model 2) and females (Model 3), there is a significant association of total AUT with *CD157* expressions for males (F (6,67) = 3.94, R^2^ = 0.31, *p*_*adj*_ = 0.003). For males, *CD157* (*coef.* = − 4.7, *t*(67) = − 2.21, *p* = 0.03). NEOO remained a significant contributor to AUT (*coef*. = 0.03, *t*(*6*7) = 2.56, *p* = 0.013) , as well as age (*coef.* = 0.60, *p* < 0.01) are significant. There are no significant results for females (Model 3).

The direction of the main effect *CD157* in males is negative although the interaction is positive. The negative direction of the CD157 main effect seems opposite to findings that OT circuitry enhances CT for females^7^. However, the presence of significant interactions suggests that the effect of CD157 on creativity is dependent on sex and CD38. To decompose the effects of CD157, we next probed the significant CD157 × Sex interaction.

**Figure 2:**
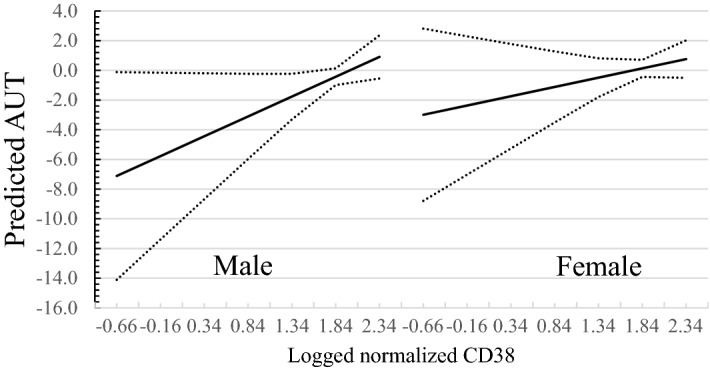
Johnson–Neyman plot of regions of significance for conditional effects of CD157 × Sex on AUT with 95% confidence intervals across log normalized CD38 levels.

Since the three-way interaction of CD38 × CD157 × Sex is marginally significant, we probed the interaction using the Johnson–Neyman method^55^. The Johnson–Neyman method shows the conditional effects of CD157 × Sex across the entire range of CD38 compared to the traditional “pick-a-point” method of probing effects at mean, 1 *SD* above and below the mean. From Fig. 2, as well as Tables S1 and S2, (in supporting material, SI), the conditional effects of CD157 expression are significant when values of logged normalized CD38 < 1.84 for males, i.e. the 95% confidence intervals did not include zero. Below CD38 < 1.84, an increase in CD157 significantly predicts an increase of AUT, a direction in line with De Dreu et al. findings of oxytocinergic contributions to creative cognition^7^.

The amount of variance in AUT explained by Model 2 for males is 31%. When we compare with the variance explained when no gene expressions were included (Model 4), the covariates of NEOO, RPM and age only explained 19%. Therefore, the inclusion of oxytocinergic gene expressions into the analysis increased the variance explained by about 12%.

### Analysis 2: relationship between AUT and dopaminergic gene expressions

In the first stage of our analyses presented above, the focus is on the oxytocinergic pathway. Table 2 demonstrates that *CD38* and *CD157* significantly correlate with CT assessments especially in males. We next examine *DRD2* and *COMT* gene expression on CT assessments.

The model was significant (F (10,136) = 4.38, R^2^ = 0.17, *p*_*adj*_ < 0.001).Contrary to expectations, there are no significant effects involving dopaminergic gene expression when males and females were pooled together (Table S3 in SI, Model 1). However, when analyzed by sex, COMT gene expression is significant (*coef.* = 1.27, *t*(64) = 2.09, *p* = 0.04) in females (Table S3 in SI, Model 3, F (6,64) = 4.06, R^2^ = 0.18, *p*_*adj*_ = 0.003).

There are significant effects of RPM (*coef.* = 0.25, *t*(136) = 2.81, *p* = 0.005) for AUT. Similarly for males (Model 2, (F (6,69) = 2.97, R^2^ = 0.19, *p*_*adj*_ = 0.014).), only NEOO (*coef.* = 0.03, t(69), *p* = 0.05) is significantly related to AUT.

### Analysis 3: relationship between AUT, oxytocinergic and dopaminergic gene expressions, RPM and openness

Finally, in the full model, we included both oxytocinergic and dopaminergic expressions and their interactions, NEOO, RPM and age. As summarized in Table S4 (SI), multiple intrasystem and intersystem interactions are significantly correlated with AUT (F(34,73) = 8.20, R^2^ = 0.34, *p*_*adj*_ < 0.001). Notably, the main effects and interactions of the gene expressions contributed were sex-dependent. There are significant effects for AUT for males (Model 2, F(18,43) = 6.60, R^2^ = 0.35, *p*_*adj*_ < 0.001) but none for females (Model 3). It must be noted that Analysis 3 is just a preliminary investigation of the interactions of the oxytocinergic and dopaminergic systems and the findings should be replicated in future studies with larger sample sizes.

## Discussion

Our results lead to a richer understanding of the neurobiology of CT and its association with trait openness and fluid intelligence. Both divergent thinking and insight problem solving were significantly associated with gene expression of *CD38* and *CD157* and their interaction in men. Surprisingly, no significant results were observed for DA expression (*DRD2*, *COMT*, *DRD2* × *COMT*), except when analyses were stratified by sex. Here, COMT gene expression was shown to be a significant contributor to AUT for females. Most importantly, the full model (including OT and DA gene expressions with multiple interactions, openness, and fluid intelligence) explained a sizable 35% of the variance in males for AUT. Overall, as hypothesised, these results are consistent with the extant literature and uniquely show that OT and DA contribute significantly to the complex creativity phenotypes using a novel gene expression strategy.

Notably, OT, the paramount human social hormone^56^, is a salient neuromodulator in these same brain regions prominent in CT and focused our attention on this nonapeptide as a likely candidate underpinning human creativity. Hence, it is crucial to examine the oxytocinergic system, the dopaminergic underpinnings of CT towards a more complete and richer understanding of this salient human trait. It should also be noted that the association between the noradrenergic system and creativity is well established^8^.

Accumulating evidence also points to an important role for dopamine neurotransmission in CT. Dopamine is implicated in motivation, emotion, personality traits, and cognitive functions that are all related directly and indirectly to creativity^57^. Genetic association studies, albeit characterized by small samples, strengthen the role of dopaminergic neurotransmission in CT^21,24^. A rich literature attests to the close relationship between DA and OT brain pathways and their joint role in often underpinning the same complex behaviors^63–61^. Notably, oxytocin neuronal fibres impinge on DA cell bodies in the ventral tegmental area and oxytocin neurons also innervate the PFC, a target of dopaminergic input^62^. Hence, it is crucial to examine the oxytocinergic system, the dopaminergic underpinnings of CT towards a more complete and richer understanding of this salient human trait.

According to a dual-process of creativity model, there are two pathways to creative thinking^24,63,64^: the flexibility and the persistence pathways. Boot and colleagues^24^ proposed an integrative review where they provide a dual DA pathway model, with the integrity of nigrostriatal-DA related to flexible processing and mesocortical-DA pathway related to persistent processing, that influences creative drive and cognition^23,65^.

Khali and colleagues especially emphasize COMT and DRD2 and their interaction in creativity^23^. This is congruent and consistent with our results using a very different methodology.

Despite the considerable heritability attributed to explaining individual differences in CT^1^, only a few candidate gene association studies have been undertaken to identify genetic loci^7,66^. A complementary strategy gaining traction that can identify jointly genetic and epigenetic contributions to complex behaviors such as CT is to implement an OMICS strategy using peripheral biomarkers, e.g.^67^. We adopt this approach and examine gene expression in saliva along with measurements of salient psychological variables. *CD38* and its homologue *CD157* (BST-1), contiguous gene duplicates on human chromosome 4 (4p15), represent a gene family that regulates cellular interactions^15^. Evidence suggests a role for both, *CD157*^19–20^ as well as *CD38* in oxytocin (OT) release and thus as putative regulators of complex behaviors^20^. Consequently, we chose to examine both homologues in the current study. A number of studies show that saliva is a reasonable target tissue for gene expression studies^68^ including behavioral disorders^69^. The use of peripheral biomarkers, and gene expression, in particular, to better understand the combined role of genes, their pathways and environment, is gaining traction^70^ in clinical psychiatry^71^, neurology^72^, drug discovery^73^, cancer treatment^74^ and psychology^53^. Saliva has been characterized as the “mirror of the body” and the “perfect medium to be explored for health and disease surveillance”^75^. Importantly, technological strides enable the stabilization of salivary RNA for downstream genomic applications^76^.

We find that the CT assessments of AUT is significantly associated with oxytocinergic (*CD38*, *CD157*) only for men but not in females. This may be an indication of oxytocin’s actions are sex-specific mediated by gonadal hormones^59,77,78^. However, no significant associations were found for *DRD2* and *COMT* gene expressions in the dopaminergic system. Notably, when analysed together, many interactions between the two systems were found to significantly associate with AUT for men).

A major limitation of the study is the small sample sizes due to missing values in gene expression, especially when both oxytocinergic and dopaminergic systems were analysed together to investigate their interactions. Nevertheless, our gene expression results extend and strengthen the findings from structural genetic studies in the literature that first surfaced the interactions of the two neurosystems for creativity and therefore, it is worthwhile to pursue future studies with larger sample sizes. Secondly, the participants carried out the AUT in 6 min due to time constraint and this may have inhibited originality to a degree. Another limitation is the limited generalizability to the general population as the participants were university undergraduates and the most of the male participants completed 1.5 years of military service. Lastly, there are a number of factors that impact creativity performance and the evidence suggests that the regulation of flexibility and stress play an important role^8^, therefore future studies are warranted to disentangle their roles.

The current investigation is one of the first to examine the neurobiological and neurochemical gene pathways in normal human cognition using a peripheral transcriptome approach. Herewith we detailed and emphasised COMT and DRD2 and their interaction in creativity, which are consistent with the DA dual pathway model. Lastly, we suggest the notion that it is opportune for a paradigm shift in neurogenetic studies of complex behavioral traits towards a more inclusive OMICS strategy that also includes the study of peripheral gene expression.

## Supplementary Information

Supplementary Tables.
