# Supplementary material for: Blending oxytocin and dopamine with everyday creativity
Source: Sci Rep. 2021 Aug 10;11:16185. doi: 10.1038/s41598-021-95724-x (PMC8355306; doi:10.1038/s41598-021-95724-x)
Supplement: Supplementary file 1 — Supplementary Tables. [file 41598_2021_95724_MOESM1_ESM.docx]

***Blending Oxytocin and Dopamine with Everyday Creativity***

*Anne Chong^1^, Serenella Tolomeo^1^, Yue Xiong^1^, Dario Angeles^2^, Mike Cheung^1^, Benjamin Becker^3^, Poh San Lai^2^, Zhen Lei^4^, Fabio Malavasi^5^, Qianzi Tang^6^, Soo Hong Chew^4^ and Richard P. Ebstein^4,7*^*

**Supplementary Material**

**Table S1**. Logged normalized values of CD38 expressions defining the Johnson-Neyman Significance Region for males.

**Table S2**. Logged normalized values of CD38 expressions defining the Johnson-Neyman Significance Region for females.

**Table S3.** The Relationship between Dopaminergic Gene Expression, AUT, Openness and Fluid Intelligence.

**Table S4.** The Relationship between Oxytocinergic and Dopaminergic Gene expressions, Openness and Fluid Intelligence on Creative Thinking (AUT).

**Table S1**. Logged normalized values of CD38 expressions defining the Johnson-Neyman Significance Region for males.

| CD38 | Margin | Delta-method Std. Err. | z | P>\|z\| | 95% Conf. Interval | |
| --- | --- | --- | --- | --- | --- | --- |
| -0.658 | -7.113 | 3.570 | -1.99 | 0.046 | -14.109 | -0.116 |
| -0.158 | -5.776 | 2.865 | -2.02 | 0.044 | -11.392 | -0.160 |
| 0.342 | -4.440 | 2.163 | -2.05 | 0.04 | -8.679 | -0.201 |
| 0.842 | -3.103 | 1.465 | -2.12 | 0.034 | -5.975 | -0.232 |
| 1.342 | -1.767 | 0.784 | -2.25 | 0.024 | -3.303 | -0.231 |
| 1.842 | -0.430 | 0.287 | -1.5 | 0.133 | -0.992 | 0.131 |
| 2.342 | 0.906 | 0.742 | 1.22 | 0.222 | -0.548 | 2.360 |

**Table S2**. Logged normalized values of CD38 expressions defining the Johnson-Neyman Significance Region for females.

| CD38 | Margin | Delta-method Std. Err. | z | P>\|z\| | 95% Conf. Interval | |
| --- | --- | --- | --- | --- | --- | --- |
| -0.658 | -2.991 | 2.961 | -1.01 | 0.312 | -8.795 | 2.812 |
| -0.158 | -2.367 | 2.378 | -1 | 0.32 | -7.027 | 2.293 |
| 0.342 | -1.742 | 1.797 | -0.97 | 0.332 | -5.263 | 1.779 |
| 0.842 | -1.117 | 1.221 | -0.91 | 0.36 | -3.511 | 1.276 |
| 1.342 | -0.493 | 0.666 | -0.74 | 0.46 | -1.799 | 0.813 |
| 1.842 | 0.132 | 0.293 | 0.45 | 0.653 | -0.443 | 0.707 |
| 2.342 | 0.757 | 0.646 | 1.17 | 0.241 | -0.509 | 2.023 |

**Table S3.** The Relationship between Dopaminergic Gene Expression, AUT, Openness and Fluid Intelligence.

|  | (1) | (2) | (3) |
| --- | --- | --- | --- |
| VARIABLES | All | Male | Female |
|  |  |  |  |
| DRD2 | 0.03 | -0.41 | -1.31 |
|  | (1.42) | (1.42) | (0.72) |
| COMT | 0.30 | -0.22 | 1.27* |
|  | (1.24) | (1.30) | (0.61) |
| DRD2 x COMT | 0.13 | 0.41 | -0.01 |
|  | (0.82) | (0.83) | (0.43) |
| Sex | 1.91 |  |  |
|  | (1.89) |  |  |
| Sex x DRD2 | -1.42 |  |  |
|  | (1.55) |  |  |
| Sex x COMT | 0.98 |  |  |
|  | (1.34) |  |  |
| Sex x DRD2 x COMT | -0.10 |  |  |
|  | (0.90) |  |  |
| Age | 0.28 | 0.37 | 0.17 |
|  | (0.16) | (0.21) | (0.24) |
| Openness | 0.02 | 0.03* | -0.00 |
|  | (0.01) | (0.02) | (0.01) |
| RPM | 0.25** | 0.25 | 0.27* |
|  | (0.09) | (0.14) | (0.11) |
| Observations | 147 | 76 | 71 |
| R-squared | 0.17 | 0.19 | 0.18 |

Legend to Table S3. Control variables used are age, sex, Openness and RPM. ***p* < 0.01; *p < 0.05

**Table S4.** The Relationship between Oxytocinergic and Dopaminergic Gene expressions, Openness and Fluid Intelligence on Creative Thinking (AUT).

|  | (1) | (2) | (3) |
| --- | --- | --- | --- |
| VARIABLES | All | Male | Female |
|  |  |  |  |
| CD38 | 52.31 | 53.76 | -114.35 |
|  | (43.43) | (39.27) | (158.00) |
| CD157 | 151.42* | 154.23* | -58.66 |
|  | (66.17) | (59.07) | (302.78) |
| CD38 x CD157 | -67.17* | -68.39* | 20.07 |
|  | (32.73) | (29.02) | (163.53) |
| DRD2 | 64.11 | 67.26 | -128.84 |
|  | (50.41) | (45.59) | (134.80) |
| CD38 x DRD2 | -29.36 | -31.13 | 61.62 |
|  | (28.15) | (25.51) | (71.01) |
| CD157 x DRD2 | -78.94 | -81.01* | 68.99 |
|  | (41.69) | (39.02) | (132.85) |
| CD38 x CD157 x DRD2 | 36.72 | 38.00 | -29.57 |
|  | (21.84) | (20.22) | (71.77) |
| COMT | 80.11 | 82.04 | -78.57 |
|  | (53.16) | (48.10) | (162.90) |
| CD38 x COMT | -34.72 | -35.55 | 37.72 |
|  | (30.13) | (27.56) | (85.30) |
| CD157 x COMT | -94.69* | -96.61** | -54.50 |
|  | (39.98) | (35.68) | (162.17) |
| CD38 x CD157 x COMT | 41.59* | 42.37* | 31.11 |
|  | (20.09) | (17.85) | (86.38) |
| DRD2 x COMT | -42.48 | -44.24 | 46.22 |
|  | (30.74) | (27.79) | (72.21) |
| CD38 x DRD2 x COMT | 19.69 | 20.65 | -22.20 |
|  | (17.30) | (15.68) | (37.77) |
| CD157 x DRD2 x COMT | 48.72* | 50.02* | -2.12 |
|  | (24.31) | (22.64) | (68.27) |
| Sex | 341.40 |  |  |
|  | (302.23) |  |  |
|  | (39.56) |  |  |
| Age | 0.28 | 0.54* | -0.21 |
|  | (0.19) | (0.25) | (0.29) |
| Openness | 0.01 | 0.01 | 0.01 |
|  | (0.02) | (0.02) | (0.03) |
| RPM | 0.33* | 0.35 | 0.18 |
|  | (0.15) | (0.17) | (0.26) |
| Observations | 108 | 62 | 46 |
| R-squared | 0.34 | 0.35 | 0.40 |

Legend to Table S4. Control variables used are age, sex, Openness and RPM. ***p* < 0.01; **p* < 0.05
